# Dermatologists’ Adherence to the Latest Recommendations for Screening of Hydroxychloroquine Retinopathy in Saudi Arabia: Cross-Sectional Study

**DOI:** 10.2196/15218

**Published:** 2019-12-19

**Authors:** Nouf Talal Mleeh, Nujood Abdulwahed Alzahrani, Jehad Osama Hariri, Hatan Hisham Mortada, Mohammed Ridha Algethami

**Affiliations:** 1 Department of Dermatology Faculty of Medicine King Abdulaziz University Jeddah Saudi Arabia; 2 Faculty of Medicine King Abdulaziz University Jeddah Saudi Arabia

**Keywords:** Saudi Arabia, dermatologist, adherence, hydroxychloroquine, retinopathy

## Abstract

**Background:**

Hydroxychloroquine (HCQ) has been used to manage many inflammatory skin conditions. Nevertheless, retinopathy continues to be its most significant adverse effect. The American Academy of Ophthalmology (AAO) recommends baseline ophthalmologic screening in the first year of HCQ treatment. However, a recent study found an inadequate awareness of the recommendations. Furthermore, limited data are available regarding the implementation of the recommendations among dermatologists.

**Objective:**

The aim of this study was to assess dermatologists’ adherence to recommendations pertaining to their current practice regarding HCQ toxicity detection.

**Methods:**

A self-administrated questionnaire was distributed between February 2 and May 4, 2018, among members of the Saudi Society of Dermatology. The questionnaire comprised demographic-related questions and questions pertaining to each physician’s routine practice about the follow-up of HCQ-treated patients.

**Results:**

A total of 76 dermatologists completed the questionnaire. We achieved a response rate of 62.54%. More than half (43/76, 56%) of the dermatologists were male. Furthermore, more than half (41/76, 53%) of them reported treating 1 to 3 patients with HCQ during the last year. Furthermore, two-thirds (47/76, 61%) of them reported screening patients before initiating HCQ treatment. Regarding follow-up recommendations, 59% (45/76) of dermatologists reported yearly after starting treatment for no-risk patients, whereas 94% (72/76) reported “yearly within 5 years of treatment” for at-risk patients. Data were considered significant at *P*<.05. All analyses were performed using SPSS, version 20 (IBM).

**Conclusions:**

Dermatologists in Saudi Arabia are not well informed about some aspects of the latest recommendations regarding screening for HCQ toxicity in terms of tests, follow-up timing, cessation of the drug, and causative agents. Therefore, we recommend conducting more studies in Saudi Arabia to determine the adherence of more physicians to the AAO recommendations. Furthermore, patient education regarding HCQ toxicity and increased patient awareness are recommended for effective and safe HCQ use.

## Introduction

### Background

Hydroxychloroquine (HCQ), a chemotherapeutic drug, inhibits the erythrocytic forms of malarial parasites with antiautophagic and immunosuppressive activities. Its main mechanism of action is inhibition of plasmodial heme polymerase [1]. HCQ, mostly used as an antimalarial drug, has been used for the management of inflammatory skin conditions for more than 50 years [2]. As it has a wide variety of uses in the treatment of many skin disorders, including cutaneous and systemic lupus, rheumatoid arthritis, and dermatomyositis [3], the use of glucocorticoid and other immunosuppressive drugs, which have serious adverse effects, has decreased [2]. Furthermore, HCQ plays an essential role in cardiovascular protection, including antithrombotic, lipid-lowering, and hypoglycemic actions [4]. HCQ’s antithrombotic effect has been attributed to a variety of mechanisms, including reduction in red blood cell aggregation, inhibition of platelet aggregation and adhesion, reduction in blood viscosity, and enhancement of antiplatelet activity [5,6]. Its lipid-lowering effects were reported in a cohort of lupus patients who showed HCQ use was associated with a 7.6% reduction in total cholesterol and a 13.7% reduction in low-density lipoprotein cholesterol over 3 months of therapy [7]. However, retinopathy debatably remains HCQ’s most feared adverse effect. The mechanism of HCQ-induced retinopathy is not fully known, but buildup in the retinal pigment epithelium could cause this condition [8]. Recently, the prevalence of HCQ-induced retinopathy was estimated to be higher than that assumed earlier (7.5%) [9]. The most important contributing factor for retinopathy appears to be the daily dose, as patients whose daily dose exceeded 5 mg/kg (actual body weight; ABW) daily had a retinopathy risk of almost 10% within 10 years of HCQ treatment [9]. Thus, ophthalmological screening for HCQ-induced retinopathy is needed, which can manifest insidiously with paracentral scotoma and subtle color vision changes, making early diagnosis challenging [10]. Thus, early screening and assessment are crucial to possibly stopping the progression of HCQ-induced retinopathy and preventing vision loss [11].

New screening guidelines published by the American Academy of Ophthalmology (AAO) recommend performing baseline ophthalmologic screening in the first year of HCQ treatment [8,12]. Therefore, HCQ could be started before the baseline assessment, which is critical, as clinical efficacy requires almost 4 to 6 months of treatment. The annual examination is recommended to start only after 5 years of HCQ use. A recent study conducted among ophthalmologists and rheumatologists to assess the adherence to the recommendations pertaining to HCQ retinopathy found an inadequate awareness of the recommendations regarding screening for HCQ toxicity [10].

### Objectives

To our knowledge, limited data are available regarding the implementation of these recommendations among dermatologists. Therefore, this study aimed to assess the adherence of Saudi dermatologists to the new recommendations in their practice regarding detection of HCQ toxicity.

## Methods

### Study Design and Data Collection

This cross-sectional study was conducted by administering a survey via email to all consultants, specialists, and senior residents of dermatology in Saudi Arabia. No sample size was calculated. Stratification considered gender and practice level. The data were collected from February 2 to May 4, 2018. All the participants were informed about the study, and those who agreed to participate were enrolled. Furthermore, the anonymity of the respondents was preserved.

### Questionnaire Variables

The questionnaire was designed by using Google forms, and responses were collected by using Google spreadsheets. The questionnaire was based on the one designed by Shulman et al [10]. Furthermore, we adapted the questionnaire on the basis of the latest recommendations for the use of HCQ in dermatologic practice [13] and on the basis of our practice of dermatology in Saudi Arabia. Only the internal consistency was measured using Cronbach alpha test. The alpha value was .88. The questionnaire comprised demographics-related questions and questions pertaining to each physician’s routine practice for the follow-up of patients treated with HCQ. The questions also addressed the physicians’ awareness of the guidelines’ recommendations, in terms of which assessments should be performed, timing of assessments, risk factors for HCQ retinopathy, and the actions to be taken if one of the screening test results is abnormal.

### Ethical Considerations

The Institutional Review Board and the Research Ethics Committee of King Abdulaziz University in Jeddah approved this study.

### Statistical Methods

The data were statistically analyzed by using descriptive statistics by Statistical Package for the Social Sciences, version 20 (IBM).

## Results

### Demographics

A total of 76 physicians completed the survey. We achieved a response rate of 62.54%. Overall, 43 (56%) participants were male. Nearly two-thirds (46/76, 60%) had medical dermatology as their specialty (Table 1).

**Table 1:** Demographic data.

Variable		Value, n (%)
**Gender**		
	Male	43 (56)
	Female	33 (43)
**Practice level**		
	Consultant	53 (69)
	Specialist	15 (19)
	Senior resident	8 (10)
**Subspecialty**		
	Medical dermatology	46 (60)
	Surgical dermatology	6 (7)
	Pediatric dermatology	2 (2)
	Other	22 (29)

### Hydroxychloroquine-Related Questions

More than half of the participants (41/76, 53%) reported treating 1 to 3 patients with HCQ during the last year. More than half of the respondents reported that they prescribed 400 mg per day of HCQ, and 25 (32%) of them knew that the correct dose is “equal to or less than 5 mg/kg ABW or 400 mg per day.” A total of 32 (42%) respondents reported prescribing HCQ between 1 and 2 years, and 24 (31%) of them reported prescribing HCQ for less than 1 year (Table 2).

A total of two-thirds of the participants (47/76, 61%) reported that they screen patients before initiating HCQ treatment, and 22 (28%) of them reported that they screened patients during the first year of HCQ treatment. The main screening tests recommended by the participants were an ocular examination (58/76, 76%) and visual field testing (41/76, 53%). Nearly half of the participants (37/76, 48%) reported performing a screening test for patients with no risk factors before initiating HCQ treatment (Table 3).

Regarding follow-up, 45/76 (59%) participants reported “yearly after starting the treatment” for no-risk patients, whereas 72 (94%) of them reported “yearly within 5 years of treatment” for at-risk patients. The main follow-up screening test was an ocular examination, performed by 59 participants (77%; Table 4).

The main risk factors reported by the participants were “previous ocular pathology” (80%) and “HCQ cumulative dose” (68%; Table 5).

The majority of the participants (63/76, 82%) reported that they never stopped the treatment because of abnormalities in screening tests; however, 21 (27%) physicians stopped treatment, as the ocular examination revealed abnormalities. A total of two-thirds of the participants (52/76, 68%) reported “follow ophthalmology recommendation” as the main action if the screening test result was abnormal (Table 6).

**Table 2:** Dermatology practice–related questions.

Variable		Value, n (%)
**In the past year, how many patients did you care for, who were treated with HCQ^a^** **?**		
	1-3	41 (53)
	4-6	13 (17)
	7-10	4 (5)
	More than 10	18 (23)
**What dose of HCQ do you usually prescribe?**		
	200 mg od	23 (30)
	200 mg bid	47 (61)
	6.5 mg/kg	2 (2)
	5 mg/kg	1 (1)
	100 mg od	2 (2)
**What is the optimal recommended dose for HCQ to reduce the risk of retinopathy? (n=72)**		
	200 mg once daily	16 (21)
	200 mg twice daily	6 (7)
	Equal to or less than 5 mg/kg of the actual body weight or 400 mg per day	25 (32)
**What is the average time your patients are currently treated with HCQ?**		
	1-2 years	32 (42)
	3-4 years	10 (13)
	Less than 1 year	24 (31)
	More than 4 years	10 (13)

^a^HCQ: hydroxychloroquine.

**Table 3:** Screening-related questions.

Variable		Value, n (%)
**Do you recommend screening tests for all patients starting treatment with HCQ^a^** **?**		
	No	6 (7)
	Sometimes	9 (11)
	Yes	61 (80)
**When do you perform the screening tests?**		
	Before initiating HCQ treatment	47 (61)
	During the first year of HCQ treatment	22 (28)
	During the first 5 years of HCQ treatment	3 (3)
	Only in patients at risk	4 (5)
**Which tests would you recommend for screening?^b^**		
	Ocular examination	58 (76)
	Color testing	13 (17)
	Visual field testing	41 (53)
	Spectral domain optical coherence tomography	19 (25)
**When would you recommend screening tests for a patient without risk?**		
	Before initiating HCQ treatment	37 (48)
	During the first 5 years of HCQ treatment	10 (13)
	During the first year of HCQ treatment	25 (32)
	Only in patients at risk	4 (5)

^a^HCQ: hydroxychloroquine.

^b^Multiple-response question.

**Table 4:** Follow-up-related questions.

Variable		Value, n (%)
**What is the recommended time of follow-up screening tests for patients without risk?**		
	Yearly, after 3 years of treatment	1 (1)
	Yearly, after 5 years of treatment	30 (39)
	Yearly, after started the treatment	45 (59)
**What is the recommended time of follow-up screening tests for patients at risk?**		
	Yearly, after 5 years of treatment	4 (5)
	Yearly, within 5 years of treatment	72 (94)
**Which follow-up tests would you recommend?^a^**		
	Ocular examination	59 (77)
	Color testing	15 (19)
	Visual field testing	43 (56)
	Spectral domain optical coherence tomography	25 (32)

^a^Multiple-response question.

**Table 5:** Factors considered by dermatologists as risk factors for retinal toxicity.

Risk factor^a^		Value, n (%)
**Age (years)**		
	<30	2 (2)
	>70	51 (67)
Renal function		36 (47)
Liver function		26 (34)
Treatment duration		51 (67)
HCQ^b^ dose		28 (36)
Cumulative HCQ dose		52 (68)
Previous ocular pathology		61 (80)
Concomitant tamoxifen use		18 (23)
Genetic factors		22 (28)

^a^Multiple-response question.

^b^HCQ: hydroxychloroquine.

**Table 6:** Abnormal screening tests.

Variable		Value, n (%)
**Have you ever stopped HCQ^a^ therapy because of an abnormal screening test?**		
	No	63 (82)
	Yes	13 (17)
**If yes, which test was abnormal?^a^**		
	Ocular examination	21 (27)
	Color testing	8 (10)
	Visual field testing	15 (19)
	Spectral domain optical coherence tomography	11 (14)
**If one of the screening tests is abnormal, what would be your next step?**		
	Decrease the dose	2 (2)
	Follow ophthalmology recommendation	52 (68)
	Stop the medication	22 (28)

^a^Multiple-response question.

## Discussion

### Hydroxychloroquine Uses and Benefits

HCQ was first discovered in the late 1960s as an antimalarial drug by Shearer and Dubois [8,10], and it has since been used as an autoimmune treatment because of its antifibrotic, antithrombotic, antidyslipidemic, and antihyperglycemic properties [11-19]. It is not fat absorbable, with an oral bioavailability of 70% and a half-life of nearly 2 months. HCQ is mostly excreted via the liver and, to a little extent, via the kidneys [20]. The drug decreases flares and the production of autoantibodies by inhibition of the toll-like receptor pathways [21,22].

The usually prescribed dose of HCQ is 4 to 6 mg/kg/day [23], and new guidelines recommend not exceeding 6.5 mg/kg of the ideal body weight (IBW) or 400 mg/day [9]. HCQ-induced retinopathy risk in optimal doses was found to be 5.0 mg/kg ABW [9], especially in thin patients [8], and 6.5 mg/kg IBW doses in obese patients, with a 400 mg/day maximum [9]. In patients receiving 5.0 mg/kg HCQ, the annual risk was lower than 1% and 4% within 10 and 20 years of treatment, respectively [4]. Patients with high daily doses exceeding 5 mg/kg ABW have been reported to be at a 10% risk for developing progressive retinopathy within 10 years of treatment initiation. In contrast, those receiving a dose of 4 to 5 mg/kg had less than 2% risk for developing progressive retinopathy within 10 years of treatment initiation [9,24-26]. Cumulative risk factors contributing to the development of retinopathy include retinal, macular or renal disease, and use of tamoxifen (risk of retinopathy increased more than 5 times the normal) [9].

Previous cohort studies have shown that median whole-blood HCQ levels >750 ng/ml and >500 ng/ml will result in significant improvement and remission [21,27-29]. In our study, more than half of the 76 dermatologists reported treating 1 to 3 patients with HCQ during the last year. More than half of the respondents prescribed a dose of 400 mg per day, and nearly 30% of them knew that the correct dose was ≤5 mg/kg ABW or 400 mg per day. Furthermore, approximately 40% participants reported prescribing HCQ between 1 and 2 years, whereas 31.6% of them reported prescribing HCQ for less than 1 year. Cox and Paterson [30] reported a study with the maximum number of responders, 325 dermatologists, and a response rate of 70%, but the dosage differed from that in our study. Nearly 90% of the patients were started on a dose of 200 mg, and only 10% of them received 400 mg of HCQ [30]. In another study, by Gilhooley et al [31], a bias was found because of the small amount of data; 36% (n=20) of the respondents in the study were dermatologists.

The AAO recommendations of HCQ dose not exceeding 6.5 mg/kg and 400 mg/day were followed by nearly 60% of rheumatologists [8]. In this study, the main follow-up screening tests were ocular examination (77.60%) and visual field testing (56.6%). However, a study conducted by Shulman et al [10] in 2017 in Tel Aviv, Israel, showed that 5% of rheumatologists and 15% of ophthalmologists were aware of baseline and follow-up evaluations of HCQ-induced retinopathy [10].

The majority of our participants stated that they recommended a screening test for all patients who were started on HCQ, and more than half of them reported recommending screening before initiating HCQ treatment, and <30% of them reported recommending screening during the first year of HCQ treatment. Compared with our study, in the study by Shulman et al [10], 85% of responders recommended baseline screening tests. It was found that nearly 30% of the rheumatologists and more than half of the ophthalmologists will delay HCQ treatment before completion of investigations [8].

Regarding follow-up recommendations for this study, nearly 60% of the participants reported annual screenings for patients without risk, whereas 94.7% of the participants reported annual screening during 5 years of treatment among high-risk patients. In the study by Gilhooley et al [31], data obtained were similar across respondents, including dermatologists and rheumatologists. A total of 43% of the respondents requested ophthalmology screening in the first year of diagnosis and then yearly, following 5 years of treatment [31]. Furthermore, 16% of both dermatologists and rheumatologists reported that a referral to ophthalmology would be recommended, with 12% requesting screening pretreatment, if visual impairment was found [31]. In contrast to the study by Shulman et al [10], in the study by Marmor et al [8], nearly 10% of responders advocated baseline follow-up investigations, following 5 years on medications in low-risk patients, whereas more than half of them proposed regular yearly investigations, and almost 30% of them maintained that usual investigations performed periodically were adequate.

The AAO guidelines highly recommend follow-up investigations after 5 years of therapy, with yearly investigations thereafter in low-risk patients and in high-risk patients who are on chronic treatment for more than 5 years, with comorbidities, elderly, with >1000 g total consumption, with >6.5 mg/kg of daily dosing. This is because the risk of retinopathy increases to 1%, following treatment for 5 to 7 years or use of cumulative dose of 1000 g among those with prolonged used of HCQ [8].

The main risk factors in this study were previous ocular pathology (reported by 80% responders), followed by HCQ cumulative dose (reported by 68% responders) and age >70 years and treatment duration (reported by 67% each). In comparison with the study by Shulman et al [10], in our study, risk factors associated with retinopathy were identified by only 4% of the responders [10].

In our study, the majority of the respondents (82%) reported that they never stopped HCQ treatment because of an abnormality, where the main test showing an abnormality was ocular examination (reported by 27%). A total of two-thirds of the respondents reported follow-up ophthalmology recommendation as the main action if the screening test was abnormal. Similarly, in the study by Shulman et al [10], nearly 80% of rheumatologists and 50% of ophthalmologists stopped HCQ treatment to some degree at one time because of uncertain retinopathy. When questioned about retinopathy, 25% of rheumatologists and approximately 5% of ophthalmologists advocated discontinuation of the medication, with no additional investigations, whereas more than half of the responders favored discontinuation of the medication with other investigations. More than 10% of rheumatologists and nearly 30% of ophthalmologists recommended adhering to the medication along with investigations [8].

Progression of HCQ-induced retinopathy can occur even if the drug is ceased [11,32]. According to a study conducted by Costedoat-Chalumeau et al [21], the relative risk of relapse was 2.5-fold higher in patients for whom classical medication was replaced with placebo than those who were maintained on classical medication.

A joint recommendation in the United Kingdom suggests that dermatologists should be familiar with HCQ-induced retinopathy screening because of the increasing number of users [33]. To the best of our knowledge, this study is the first of its kind to be conducted in Saudi Arabia. This study recommends further studies to assess the factors responsible for following ophthalmologic recommendations by the dermatologists, and this study recommends a further randomized controlled trial to compare the different ophthalmologic screening processes.

### Limitations

Although our sample size was much larger than any reported article in the field, and the study has reached its aim, there were some limitations that need to be highlighted. First, our study was a cross-sectional study, and it covered a short interval time, and there is a possibility that the responses are not representative of all health care providers. Second, response bias may be possible, which was particularly affected by refusal of some to participate or failure to complete the questionnaire, and this could be attributed to lack of interest and time, which resulted in their exclusion from the study. Finally, there was a lack of studies in the literature in the field.

### Conclusions

We conclude that dermatologists in Saudi Arabia are not well informed about the latest AAO recommendations regarding screening for HCQ toxicity in terms of tests, follow-up timing, cessation of the drug, and causative agents. Furthermore, we found that these dermatologists are somewhat knowledgeable about the latest recommendations pertaining to HCQ treatment; however, there was a bias in this study because of the small number of responders. The benefits of continuing treatment with HCQ should outweigh the risks of treatment discontinuation, and risk factors that may exacerbate toxicity should be considered. We recommend conducting more studies in Saudi Arabia to evaluate the adherence of a greater number of physicians. Education of patients is also necessary for effective and safe treatment with HCQ. Patients are advised to schedule ophthalmology visits if there are any visual changes or any new comorbidities, including renal/liver diseases or significant weight changes.

## Abbreviations

AAO: American Academy of Ophthalmology
ABW: actual body weight
HCQ: hydroxychloroquine
IBW: ideal body weight
